# Reversion to Normal of *FMR1* Expanded Alleles: A Rare Event in Two Independent Fragile X Syndrome Families

**DOI:** 10.3390/genes11030248

**Published:** 2020-02-26

**Authors:** Elisabetta Tabolacci, Roberta Pietrobono, Giulia Maneri, Laura Remondini, Veronica Nobile, Matteo Della Monica, Maria Grazia Pomponi, Maurizio Genuardi, Giovanni Neri, Pietro Chiurazzi

**Affiliations:** 1 Fondazione Policlinico Universitario “A. Gemelli” IRCCS, Istituto di Medicina Genomica, Università Cattolica del Sacro Cuore, 00168 Roma, Italia; elisabetta.tabolacci@unicatt.it (E.T.); roberta.pietrobono@unicatt.it (R.P.); giuliamaneri@gmail.com (G.M.); veronicanobile88@gmail.com (V.N.); maurizio.genuardi@unicatt.it (M.G.); pietro.chiurazzi@unicatt.it (P.C.); 2 Fondazione Policlinico Universitario A. Gemelli IRCCS, UOC Genetica Medica, 00168 Roma, Italia; laura.remondini@policlinicogemelli.it (L.R.); mariagrazia.pomponi@policlinicogemelli.it (M.G.P.); 3 UOC Genetica Medica e di Laboratorio, Azienda Ospedaliera di Rilievo Nazionale “A. Cardarelli”, 80100 Napoli, Italia; matteo.dellamonica@aocardarelli.it; 4 JC Self Research Institute, Greenwood Genetic Center, Greenwood, SC 29646, USA

**Keywords:** *FMR1* gene, fragile X syndrome, CGG instability, CGG reversion, genetic test

## Abstract

Fragile X syndrome (FXS) is mostly due to the expansion and subsequent methylation of a polymorphic CGG repeat in the 5’ UTR of the *FMR1* gene. Full mutation alleles (FM) have more than 200 repeats and result in *FMR1* gene silencing and FXS. FMs arise from maternal premutations (PM) that have 56–200 CGGs; contractions of a maternal PM or FM are rare. Here, we describe two unaffected boys in two independent FXS families who inherited a non-mosaic allele in the normal and intermediate range, respectively, from their mothers who are carriers of an expanded CGG allele. The first boy inherited a 51 CGG allele (without AGG interruptions) from his mother, who carries a PM allele with 72 CGGs. The other boy inherited from his FM mother an unusual allele with 19 CGGs resulting from a deletion, removing 85 bp upstream of the CGG repeat. Given that transcription of the deleted allele was found to be preserved, we assume that the binding sites for *FMR1* transcription factors are excluded from the deletion. Such unusual cases resulting in non-mosaic reduction of maternal CGG expansions may help to clarify the molecular mechanisms underlying the instability of the *FMR1* gene.

## 1. Introduction

Fragile X syndrome (FXS, OMIM #300624) is the most common monogenic cause of intellectual disability (ID) and belongs to the Fragile X-related disorders [1], a group of genetic conditions that includes also fragile X-associated tremor/ataxia syndrome (FXTAS, OMIM #300623) [2] and fragile X-associated primary ovarian insufficiency (FXPOI, OMIM #311360) [3]. All these conditions are due to the expansion of a polymorphic CGG repeat sequence located in the 5′ UTR of the *FMR1* gene [4]. Depending on the size of the CGG repeat, four different classes of alleles may be defined [5]: (1) normal alleles with 6–44 CGGs, with the most common alleles having 29–30 repeats, that are stably transmitted to the offspring; (2) intermediate or “gray zone” alleles with 45–55 repeats, that might be unstable, especially when AGG interruptions are absent [6,7,8]; (3) premutation (PM) alleles with 56–200 CGGs, which are associated with FXTAS and FXPOI; (4) full mutation (FM) alleles with more than 200 CGG repeats (up to 1000 and more), typically inducing local DNA hypermethylation and *FMR1* gene silencing. When a PM is maternally transmitted, it may expand to a full mutation (FM) allele, resulting in absence of the FMRP protein and consequently in FXS.

Both expansions and contractions upon meiotic transmission and during mitotic divisions have been described in FXS families, with expansions outnumbering contractions by 10:1 in both humans and in PM (knock-in) mouse models [9,10]. Size instability contributes to the mosaicism frequently seen in carriers of PM and FM alleles. PM alleles usually undergo only small expansions/contractions when transmitted by a father and male PM carriers are not considered to be at risk of transmitting a FM allele to their daughters [11]. Large expansions from PM to FM are apparently transmitted only through maternal meiosis [12]. In addition to the sex of the transmitting parent, the main risk factors for instability are: the number of repeats, the number (and position) of AGG interruptions, and maternal age [7]. Unstable alleles probably arise from stable ones after the loss of AGG interruptions [13]. For example, the presence of two interspersed AGGs in PM alleles of 70–90 repeats associates with a 60% risk reduction of transmitting a FM to the next generation [6]. On the other hand, contractions, observed both after maternal and paternal transmission, do not seem to be influenced by the presence of AGG interruptions [8]. Mismatch (MMR) and base excision (BER) repair mechanisms, as well as slipped strand mispairing (SSM) or polymerase slippage during DNA replication, appear to be involved in contraction, as well as in expansion events [14]. Contractions may arise from the difficulty of DNA polymerase in progressing through the repeats under conditions where rapid cell division is required. This may explain why FM alleles are not usually seen in the sperm of FXS males [15]. These events are frequently associated with the loss of AGG interruptions on maternal but not paternal transmission [8]. Understanding the mechanisms and pathways that lead to contractions (or to instability in general) is very important for devising strategies aimed at blocking further expansions. In a proof of principle experiment, CGG repeats were removed using the CRISPR/Cas9 technique, reactivating *FMR1* transcription [16]. Unfortunately, this technology is not mature yet for in vivo applications due to the risk of off-target effects and the difficulty of reaching target cells in the brain.

We here describe two independent FXS families in which two unaffected boys inherited a non-mosaic *FMR1* allele in the normal/gray-zone range derived from the expanded allele of their mothers, respectively. One CGG reversion was diagnosed postnatally in a normal child, while the second case was accidentally detected during prenatal diagnosis on chorionic villi as well as amniocytes and confirmed after the child’s birth on blood leukocytes. These rare events, that apparently occurred during maternal meiosis (i.e., pre-zygotically), may be challenging to interpret, particularly in the context of a prenatal diagnosis in the absence of phenotypic information, since the presence of an undetectable expanded allele in mosaic form cannot be excluded with certainty. A question remains unanswered: will a contracted allele, derived from an expanded one, behave like a stable allele or could it expand again when transmitted to the next generation?

## 2. Patients and Methods

### 2.1. Patients

**Figure 1:**
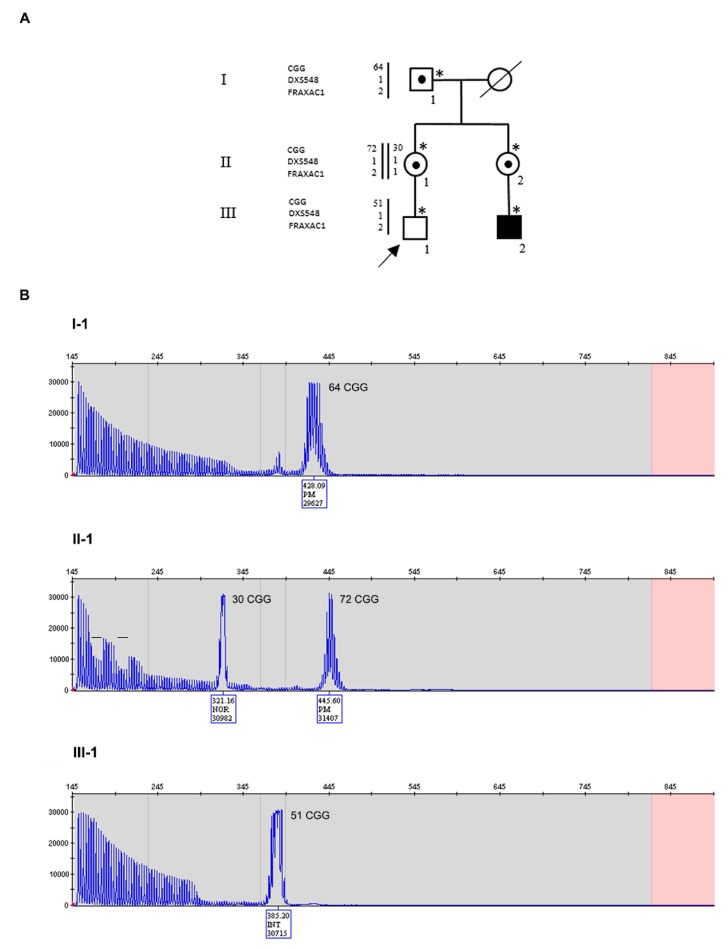
Pedigree of Family 1 with haplotype segregation within the *FMR1* locus and CGG sizing through PCR. (**A**) Black square (III-2) indicates the affected FXS boy, carrier of a methylated full mutation MFM; a dotted square and circles indicate carriers of PM. Individuals tested for the *FMR1* expansion are indicated with an asterisk near the symbol. On the left side, the polymorphic markers (CGG, *DXS548* and *FRAXAC1*) are reported. Note that *DXS548* was not informative. (**B**) Capillary electrophoresis of the fluorescent PCR used for CGG sizing of the maternal grandfather I-1 (upper panel), of the mother II-1 (middle panel), and of the proband III-1 (lower panel). In each panel, the CGG repeat number was estimated from the size of the PCR products. The proband had an intermediate size allele of 51 CGGs derived from the expanded allele of his mother, without any apparent sign of mosaicism. I-1 was PM carrier of a 64 CGG repeats allele; II-1 was heterozygous for a normal allele of maternal origin and a paternal PM of 72 repeats. Bars on the capillary electrophoresis of II-1 indicated the presence of two canonical interspersed AGGs on her normal allele, since the expanded allele has no interruptions in I-1 as well as in III-1.

**Figure 2:**
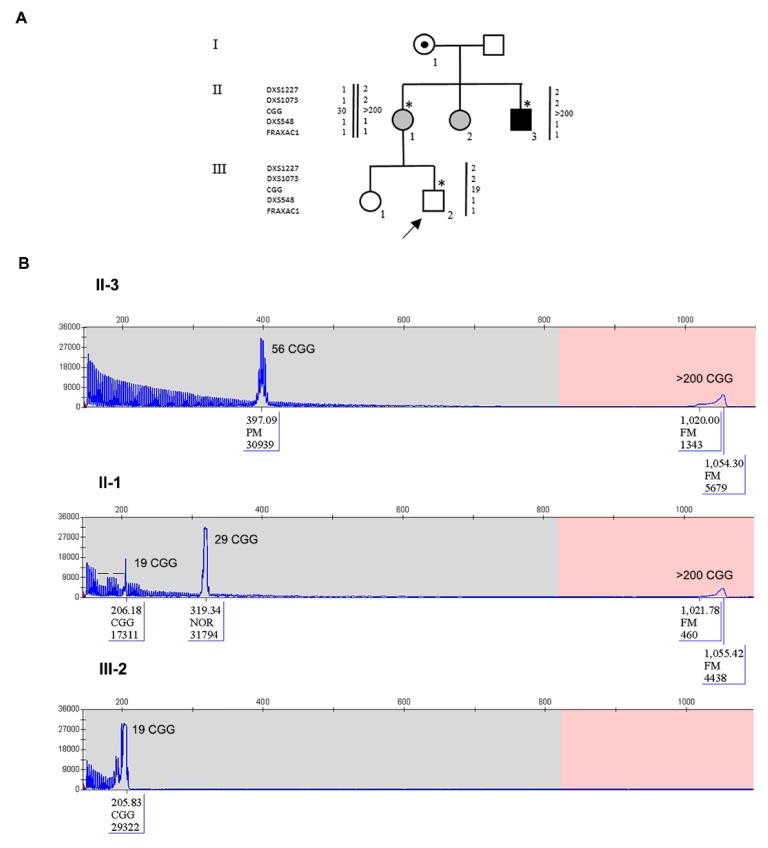
Pedigree of Family 2 with haplotype segregation within the *FMR1* locus and CGG sizing through PCR. (**A**) Black square (III-3) indicates the affected FXS individual, carrying a MFM; grey circles indicate FM carrier females. Individuals tested for the *FMR1* expansion are indicated with an asterisk near the symbol. On the left side, the polymorphic markers (CGG, *DXS1227*, *DXS1073*, *DXS548* and *FRAXAC1*) are reported. Note that *DXS548* and *FRAXAC1* were not informative. (**B**) Capillary electrophoresis of the fluorescent PCR for CGG sizing of the proband’s maternal uncle II-3 (upper panel), of the mother II-1 (middle panel), and of the proband III-2 (lower panel). In each panel, the CGG repeat number was estimated from the size of the PCR products. In III-2, the allele is shorter than the normal range without signs of interspersed AGGs and no sign of mosaicism. On the other hand, II-3 is a mosaic carrier of a small PM of 56 CGGs and a FM while II-1 was eventually found to be a mosaic between a FM and a contracted allele similar to that inherited by her child (III-2). The proband had the reversed allele with the 85 bp deletion followed by 19 CGGs derived from his mother. Bars on the capillary electrophoresis of II-1 indicated the presence of two canonical interspersed AGGs on the normal 29 CGG repeat allele.

Participants provided written informed consent for the molecular analysis performed in this study as well as for sharing clinical information. The study protocol was approved by the Ethic Committee of the Catholic University of Rome (prot. N. 9917/15 and prot.cm 10/15). Pedigrees of Family 1 and Family 2 are reported in Figure 1A and Figure 2A, respectively, in which all family members studied by molecular analysis are indicated. DNA was extracted using standard salting-out protocol from peripheral blood leukocytes of individuals of both families and from chorionic villi sample (CVS) and amniocytes obtained prenatally from individual III-2 of Family 2.

### 2.2. CGG Sizing and Methylation Analysis

CGG sizing was assessed by AmplideX^®^ PCR assay (Asuragen, Austin, TX, USA), a sensitive and efficient amplification method [17]. This CGG Repeat Primed (RP) PCR protocol uses three primers and fragment sizing on a Genetic Analyzer (Thermo Fisher), allowing detection of AGG interruptions within the *FMR1* allele. PCR reagents include gene-specific and CGG primers, a polymerase mix buffer for amplification of the CGG repeat region in the *FMR1* gene and a ROX 1000 Size Ladder for sizing by capillary electrophoresis. Amplified products were visualized by capillary electrophoresis (3500xL Genetic Analyzer, Thermo Fisher, Waltham, MA, USA) and separated using POP-7^TM^ polymer (Thermo Fisher), following manufacturer’s instructions. The size of the PCR products has been converted to the estimated number of CGG repeats using size and mobility conversion factors. The reference sequence of the *FMR1* gene used for sizing the CGG repeat is LRG_762 (identical to NG_007529.2).

Evaluation of genomic DNA methylation was performed through MS-MLPA on prenatal diagnosis samples and peripheral blood leukocytes of III-2 (Family 2), using the specific assay for *FMR1-AFF2* locus in Xq27.3 (code ME029B2), following the manufacturer’s protocol (MRC Holland).

### 2.3. Haplotype Analysis

Segregation study was performed on all family members indicated in Figure 1A and Figure 2A. PCR amplification of the polymorphic markers *DXS548* (forward 5′-AGA GCT TCA CTA TGC AAT GGA ATC and reverse 5′FAM-GTA CAT TAG AGT CAC CTG TGG TGC) and *FRAXAC1* (forward 5′-GAT CTA ATC AAC ATC TAT AGA CTT TAT T and reverse 5′VIC-AGG CTT GGA GTG CAG TGG GCA ATC T), mapping both in Xq27.3, *DXS1227* in Xq27.2 (forward 5′-AAA ACC AAG CTG TTT ACT GCT CC and reverse 5′FAM- ACT CAT CAG ACA TAG GTG GGG AA) and *DXS1073* in Xq28 (forward 5′-GGC TGA CTC CAG AGG C and reverse 5′FAM-CCG AGT TAT TAC AAA GAA GCA C) was carried out with fluorescent primers. Alleles were resolved on capillary gel electrophoresis using an ABI3130 sequencer and analysed with GeneMapper v4.0 (Thermo Fisher).

### 2.4. Sequencing Analysis

Genomic DNA from CVS and peripheral blood leukocytes of III-2 (Family 2) was amplified using primers “c” and “f” [18], that span the CGG repeat region (LRG_762, the same of NG_007529.2). Amplification products were sequenced in both directions with BigDye Terminator v3.1 Cycle Sequencing kit (Life technologies, Carlsbad, CA, USA, 4336917) and separated on a 3130 Genetic Analyzer (Life technologies).

### 2.5. FMR1-mRNA Quantification

Total RNA was extracted from CVS and amniocytes of III-2 (Family 2) as well as two unaffected matched control males by standard Trizol protocol (Life biotechnologies). 500 ng of total RNA were retro-transcribed into cDNA by SensiFAST cDNA Synthesis kit (Bioline, Memphis, TN, USA), according to the manufacturer’s instructions. For a relative quantification of *FMR1* transcript using ABI7900HT (Life technologies), the following pre-developed TaqMan^®^ assays were employed: *FMR1* (Hs.PT.58.2579089, IDT) and *GAPDH* (glyceraldehyde-3-phosphate-dehydrogenase) (Hs.PT.39a.22214836, IDT), the latter being constitutively expressed in every cell and thus used as endogenous control. The cycle parameters were: 2 min at 50 °C and 10 min at 95 °C, followed by 40 cycles with 15 seconds at 95 °C (denaturation) and 1 min at 60 °C (annealing/extension). The relative quantification of target transcript vs. endogenous transcript was calculated as follows: 2^−(ΔCt(*FMR1*))−ΔCt(*GAPDH*))^ = 2^−ΔΔCt^, where ΔCt is the difference (Ct(*FMR1*)–Ct(*GAPDH*)) and Ct is the cycle at which the detected fluorescence overcomes the threshold.

## 3. Results

### 3.1. Family 1

The proband III-1 (Figure 1A) is a normally developing 11-year-old boy, whose mother (II-1) and maternal aunt (II-2) are PM carriers. The aunt has a child (III-2) affected by FXS, carrier of a methylated FM (MFM). The PM alleles derive from individual I-1, who carries a PM allele of 64 triplets with no AGG interruptions (Figure 1B, upper panel). He started to manifest dysarthria at 55 years and then ataxia, leading to a diagnosis of FXTAS at the age of 66 years. The proband’s mother (II-1) had a normal allele of 30 repeats with two interspersed AGGs and a PM of 72 CGGs without AGGs (Figure 1B, middle panel). She developed FXPOI with menopause occurring at 37 years. Her sister (II-2) had a normal allele of 30 CGGs and a premutated one of 68 CGGs (not shown). Unexpectedly, individual III-1 was found to carry a gray-zone allele of 51 CGGs without AGG interruptions and no evidence of size mosaicism in peripheral leukocytes (Figure 1B, lower panel). Segregation analysis with CGG sizing and two polymorphic markers flanking the *FMR1* locus (*DXS548*, not informative, and *FRAXAC1,* informative) was also performed and the results are reported in Figure 1A. Comparing the maternal grandfather I-1 with the proband and his mother, we inferred that the X chromosome of III-1 was inherited from I-1, after undergoing contraction during maternal meiosis.

### 3.2. Family 2

**Figure 3:**
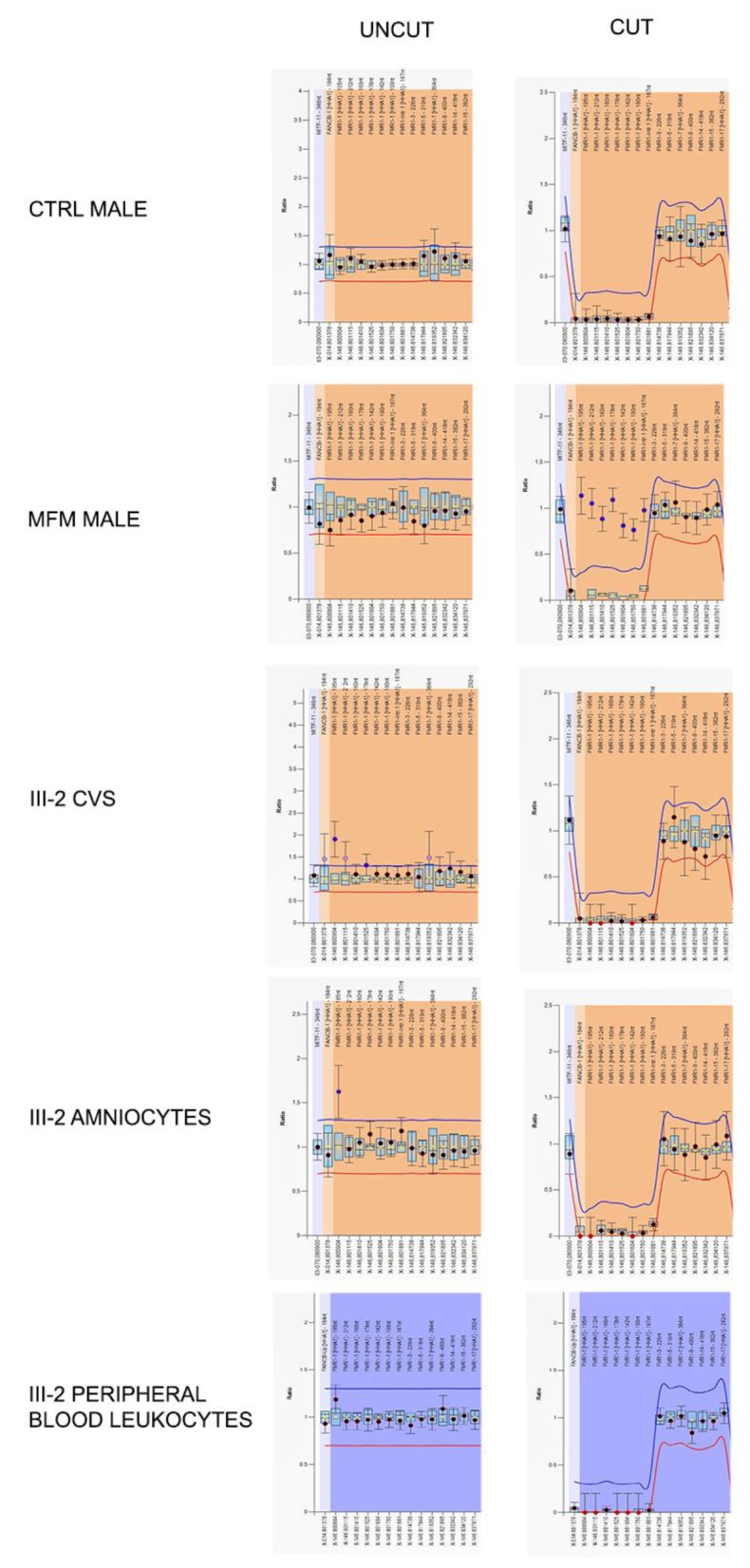
Graphical representations of MS-MLPA analysis at the *FMR1* locus performed on Family 2 proband’s DNA during prenatal and postnatal diagnoses. Only probes of the *FMR1* locus are shown. Left panels represent the results before the digestion with methylation-sensitive *Hha*I enzyme, while the right ones are those after the cut. The *Hha*I-sensitive probes are indicated in square brackets. In control male (after *Hha*I cut) the genomic DNA methylation drops to zero due to the absence of *FMR1* methylation and thus the enzyme could cut the DNA. On the contrary, in a MFM male, *FMR1* probes remained uncut due to the presence of DNA methylation. In proband III-2, *FMR1* methylation levels fall after *Hha*I digestion, because the *FMR1* locus is unmethylated as in a normal control male. *FMR1* gene was unmethylated both in prenatal (CVS and amniocytes) and in postnatal (peripheral blood leukocytes) samples.

**Figure 4:**
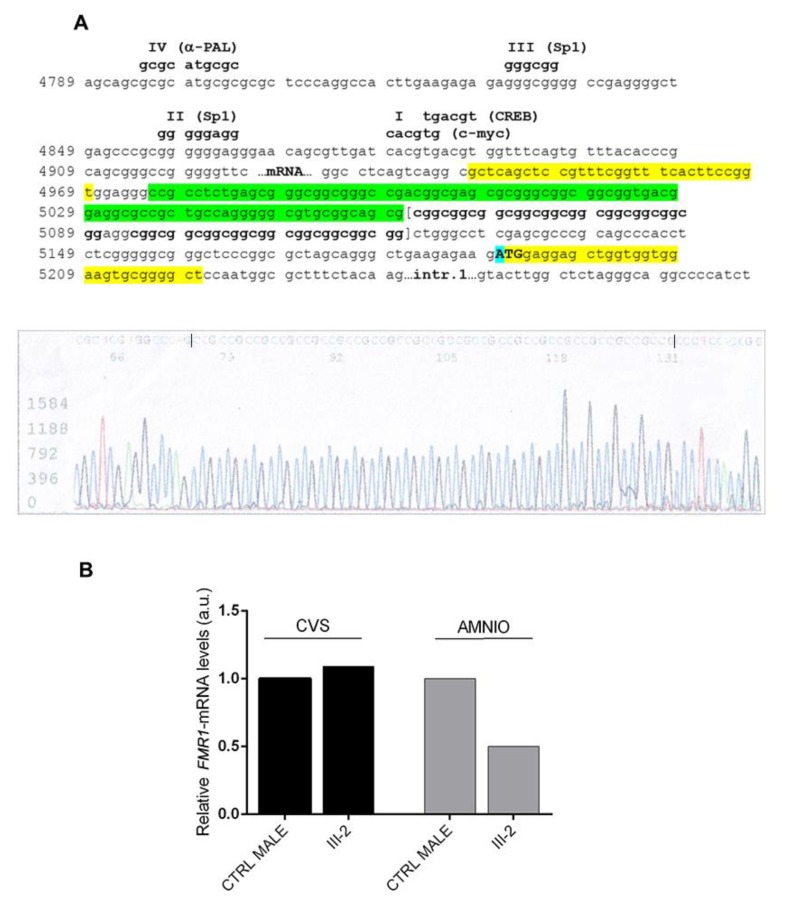
Sequencing analysis of the *FMR1* promoter region and *FMR1* expression levels in proband III-2 from Family 2. (**A**) The upper panel details the sequence of the *FMR1* promoter region (according to reference sequence NG_007529.2). The 85 bp deleted region (nucleotides 4975–5060) in proband III-2 is indicated in green and only 19 CGGs were visible following the deletion. Highlighted in yellow are the sequences of the “c” and “f” primers used to sequence the proband’s genomic DNA. The ATG (first codon) is indicated in upper case and the A (c.1), highlighted in blue, corresponds to position 5190 of NG_007529.2. The four known footprints (IV, III, II and I) are indicated above the DNA sequence with the transcriptional factors that bind them. The lower panel shows the electropherogram of III-2 genomic sequence using a reverse primer (therefore CCG repeats can be observed). (**B**) Relative quantification of *FMR1*-mRNA by RT-PCR on CVS and amniocytes of proband III-2 and matched controls (CTRL). In CVS *FMR1*-mRNA was expressed at the same level of a control male, while in amniocytes *FMR1* expression levels were approximately 50% lower in III-2 compared to the control. Values reported on the *y*-axis represent relative transcriptional levels normalized to the control sample, arbitrarily set to 1.

The pedigree of Family 2 is reported in Figure 2A. The proband III-2 underwent *FMR1* molecular analysis during prenatal diagnosis. His mother (II-1) was known to be carrier of a normal allele and a MFM allele with an expansion of 1.2–1.5 kb (approximately 430–530 CGGs), originally detected by Southern blot (not shown). His maternal uncle II-3 is affected by FXS and carries a MFM of 1.5–2.7 kb in size (as estimated by Southern blot, not shown), corresponding to approx. 530–930 CGGs. His maternal aunt II-2 has a FXS diagnosis and carries an expansion in the FM range assessed elsewhere (not shown). The expansion arose from maternal grandmother I-1, who was carrier of a PM, as referred by II-1. During the first pregnancy of II-1, a prenatal diagnosis through CVS was performed elsewhere and resulted in a normal female (III-1). For the second pregnancy, CVS was again chosen and performed at 13 weeks of gestation. The fetal karyotype was normal male (46,XY) and *FMR1* molecular analysis was performed by PCR and MS-MLPA to estimate the size and the methylation status. Surprisingly a single peak corresponding to a completely unmethylated allele, smaller than the expected size, was detected, without evidence of mosaicism. This allele was then sequenced and a deletion of 85-bp immediately upstream the repeat, followed by 19 CGGs with no AGG interruptions was observed. Haplotype analysis (*DXS1227* and *DXS1073* were informative, while *DXS548* and *FRAXAC1* were not informative) confirmed that the fetus had inherited from his mother the same fragile X chromosome present in his affected maternal uncle II-3 (Figure 2A). Finally, we performed RT-PCR analysis of the *FMR1* transcript on RNA extracted from CVS and found levels of mRNA comparable to those of matched control cells. The same findings were further confirmed through amniocentesis at 19 weeks of gestation. The mother completed pregnancy and the boy was born and appears to be developing normally at the age of 3 years. Recently, molecular analysis of *FMR1* on peripheral blood leukocytes confirmed the results obtained prenatally. PCR analysis of the *FMR1* locus in the proband’s mother and maternal uncle allowed a more precise estimate of the CGG number (Figure 2B). During this analysis, a small peak was observed in II-1 corresponding to the contracted allele inherited by the proband, which could not be detected by the initial Southern blot analysis. We could thus conclude that individual II-1 is a mosaic between a FM allele and a contracted allele with 19 uninterrupted CGG repeats, that was passed on to her son, while her brother II-3 is mosaic for a FM and a PM with 56 CGGs (Figure 2B). Methylation analysis of the *FMR1* locus performed in prenatal and postnatal samples of the proband is summarized in Figure 3 and shows that the gene promoter is unmethylated in II-1. Sequence analysis indicated that the deleted region extends from position 4975 to 5060 of *FMR1* reference sequence NG_007529 and includes also part of the CGG repeat (Figure 4A). Because the DNA binding sites for the main transcription factors were not included in the deleted region, transcription levels of the *FMR1* gene remained substantially in the normal range with lower expression levels in amniocytes compared to CVS (Figure 4B).

## 4. Discussion

Reversion to normal and intermediate sizes of expanded *FMR1* alleles is reported in two independent FXS families. Two females, carriers of an expanded allele, transmitted an allele of normal and intermediate size to their respective unaffected male offspring. CGG instability within the *FMR1* locus occurs mainly from PM to FM size, while contractions from PM or FM to normal (or rarely PM) have exceptionally been reported. Expansion and contraction can be considered two faces of the same coin, both resulting from the instability of the CGG repeat sequence. These two apparently different outcomes may derive from common mechanisms, such as mismatch repair, base-excision repair and slipped strand mispairing/polymerase slippage occurring during DNA replication or repair. The main risk factor for instability is represented by the number of AGG interruptions, usually found every nine or 10 CGG repeats in normal alleles [6]. Despite the fact that contraction events were reported to be independent of AGG presence [8], in the present cases, the two reverted alleles derived from maternal expanded CGG repeats without AGG interruptions. In other published cases of contracted alleles, AGG interruptions were absent except for one female who inherited a 54 CGG grey zone allele by her mother, carrier of a 93 CGG PM [19]. In a recent paper, a model for CGG instability has been refined studying 5508 transmissions alleles from males and females [20]. Instability of the *FMR1* repeat is influenced by the size of the repeat itself, its internal structure and the sex of the transmitting parent. In particular loss of AGG interruptions occurred exclusively during contraction events of maternal PM alleles, as in the described cases (eight events of AGG loss in 32 maternal contractions vs. no events of AGG loss in 68 paternal contractions). In both our probands (III-1 in Family 1 and III-2 in Family 2) and in all studied samples the contracted alleles were detected in an apparently non-mosaic state, although we cannot exclude that allele size mosaicism may exist in other tissues. Contracted alleles detected in FXS families in a non-mosaic state have already been reported by us, in an unaffected boy who inherited an allele of 43 CGGs derived from a large PM (around 190 CGGs) carried by his mother [21]. Similar reverted and non-mosaic alleles have been described in FXS families both in unaffected females [19,22] and in unaffected males [23,24]. Contracted alleles in the range of normality have also been detected along with expanded ones in FXS mosaic males, such as in the four affected boys reported by Maia et al. [25], who inherited the normal allele by their FM or PM mother, or as in a monozygous male twin, who derived the normal allele from his PM mother [26]. These latter cases are probably due to somatic instability of the CGG expansions in the range of PM or FM, which is well documented [27,28], while information regarding unstable normal alleles is limited [29].

In Family 1, the contracted allele of 51 CGGs found in the proband was absent in his mother, who carried a PM of 72 repeats. This finding suggests that the CGG contraction event was *de novo*, likely resulting from an intramolecular event involving the expanded tract either in the maternal germ line or (less likely) during early embryogenesis. The regulatory regions flanking the CGG repeats were preserved, thus allowing normal *FMR1* gene activity and normal phenotype. In this family the absence of interspersed AGGs in the expanded allele was most likely a factor contributing to the instability that led to the reduction. The maternal grandfather had a 64 CGG repeat, which expanded to 72 repeats in his daughter II-1 and to 68 in daughter II-2. Both PM alleles continued to show instability: in one case a canonical expansion to MFM caused FXS in individual III-2, while in the other case a contraction rescued individual III-1 from being affected by FXS or at risk for FXTAS.

In Family 2, the proband’s mother (II-1) was heterozygous for a normal *FMR1* allele of 29 CGGs (with two typical AGG interruptions) and for a FM allele. Our subsequent re-evaluation by PCR discovered an atypical shorter-than-normal allele (harboring the 85 bp deletion and 19 uninterrupted CGG repeats), which had gone undetected by Southern blotting. The expanded allele derived from grandmother (I-1), reportedly a PM carrier. Although we could not analyze the DNA of I-1, it is reasonable to assume that the 85 bp deletion/19 CGGs-contracted allele arose *de novo* in II-1 during early embryogenesis since it represents only a very minor proportion of her leukocyte DNA. This unusual allele passed through the maternal germ line to her unaffected son III-2. We first noted it during prenatal diagnosis (performed on chorionic villi and amniocytes) and then confirmed after birth in peripheral blood leukocytes. Because of its unusual size and since it had been detected during a prenatal diagnosis in a male fetus, this allele has been analyzed for methylation status and sequenced. No sign of methylation was detected by MS-MLPA, but an 85 bp deletion was detected immediately upstream of the repeat region followed by 19 uninterrupted CGG triplets. A similar case had been reported more than twenty years ago in a female who was partially hemizygous because of a large deletion of one X chromosome which included the *FMR1* locus, while the other X chromosome had a microdeletion in the *FMR1* gene including all CGG repeats and 97 bp of flanking sequences, leaving both the transcription start site and the translation initiation site intact [30]. While in this case both *FMR1* alleles are deleted, though to different extent, suggesting a meiotic unequal crossing-over event, the 85-bp/19 CGG deletion identified in the mother of III-2 should derive from a post-zygotic intramolecular rearrangement during early embryogenesis since it is found in mosaic state in both her blood and gametes. This observation underlines once more the critical period of initial embryonic development when the speed of mitotic divisions is likely to facilitate replication errors.

*FMR1* transcription in CVS cells of proband III-2 was similar to that of a matched control male and just slightly lower in amniocytes, possibly due to tissue variability in *FMR1* gene expression (Figure 4B). During prenatal diagnosis we could not exclude with certainty the presence of an undetected expanded allele of the *FMR1* gene, possibly in the range of a FM. Only after birth, we witnessed the normal development of the child and confirmed in peripheral blood leukocytes the molecular results obtained in CVS cells and amniocytes. One remaining question concerns the stability of the contracted alleles in future generations. Given its very small size, there is no special reason to consider the 19 CGG allele unstable through either maternal or paternal meiosis, even though it lacks AGG interruptions. On the other hand, the 51 CGG allele of Family 1 is likely to be unstable, given the absence of interspersed AGGs. Actually, for intermediate-size alleles such as this with 51 CGGs, transmission of the repeat through males was found to be less stable than that through females [11]. This pattern differs from that seen for PM alleles: paternally transmitted alleles are far more stable than maternally transmitted alleles.

Unusual and rare cases, such those reported here, deserve special attention because they may help to clarify the molecular mechanisms underlying the instability of the *FMR1* gene.
